# Supplementary figures and images for: Transcriptomic Analysis Reveals Patterns of Expression of Stage-Specific Genes in Early Apis cerana Embryos
Source: Genes (Basel). 2025 Feb 3;16(2):187. doi: 10.3390/genes16020187 (PMC11855871; doi:10.3390/genes16020187)

Total Isoform Expression Comparison

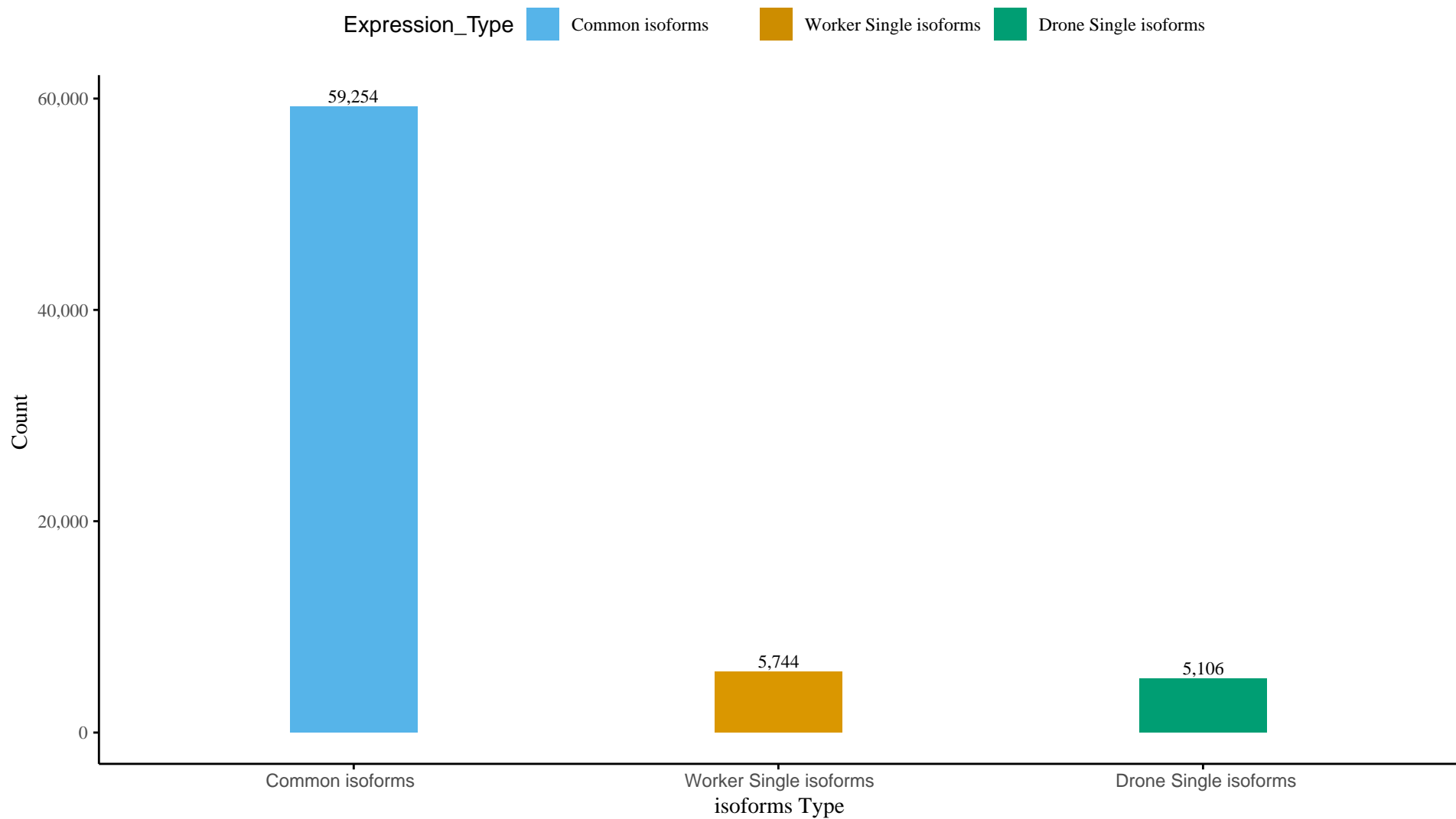

Supplement: Supplementary file 1 [file genes-16-00187-s001.zip › Supplemental Figure S1.pdf]

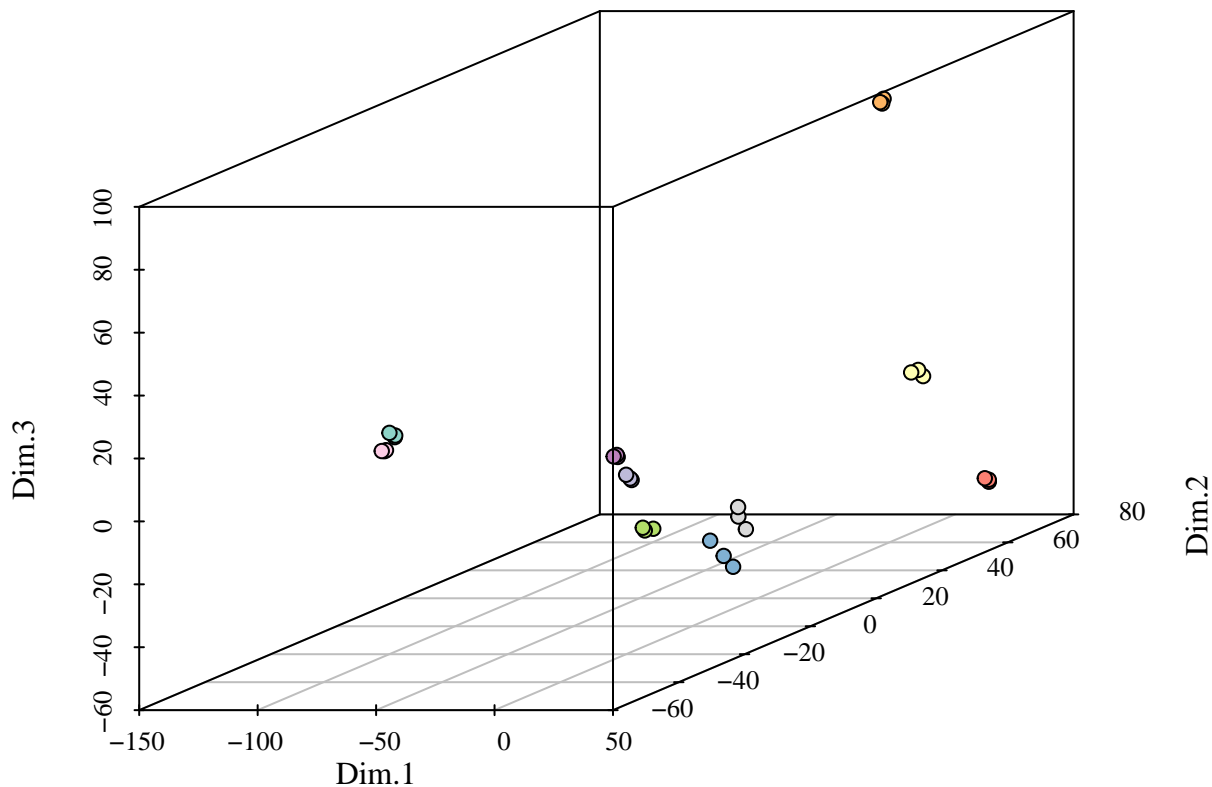

● G-M1 ● G-M2 ● G-M3 ● G-M4 ● G-M5 ● X-M1 ● X-M2 ● X-M3 ● X-M4 ● X-M5

Supplement: Supplementary file 1 [file genes-16-00187-s001.zip › Supplemental Figure S2.pdf]

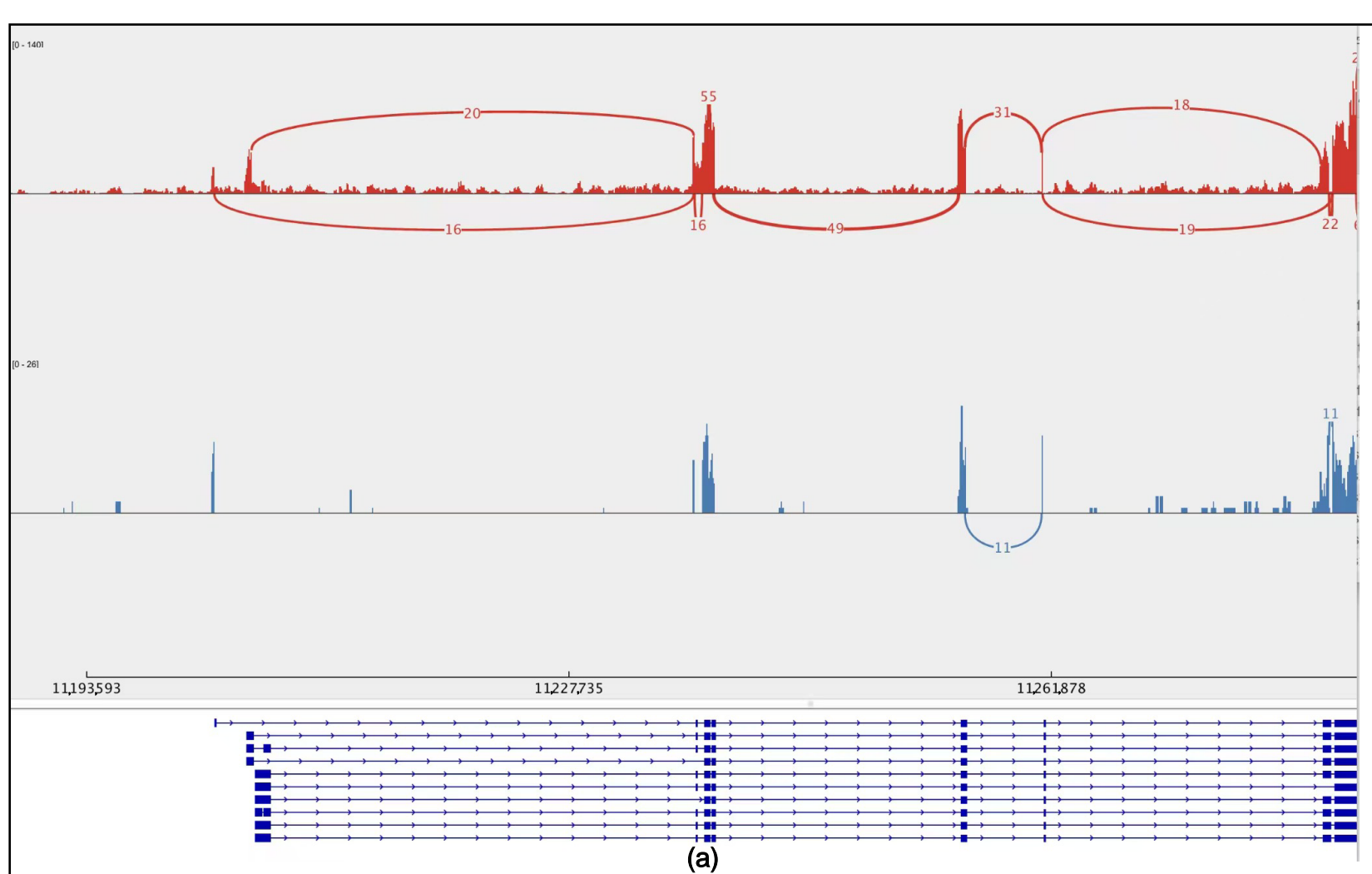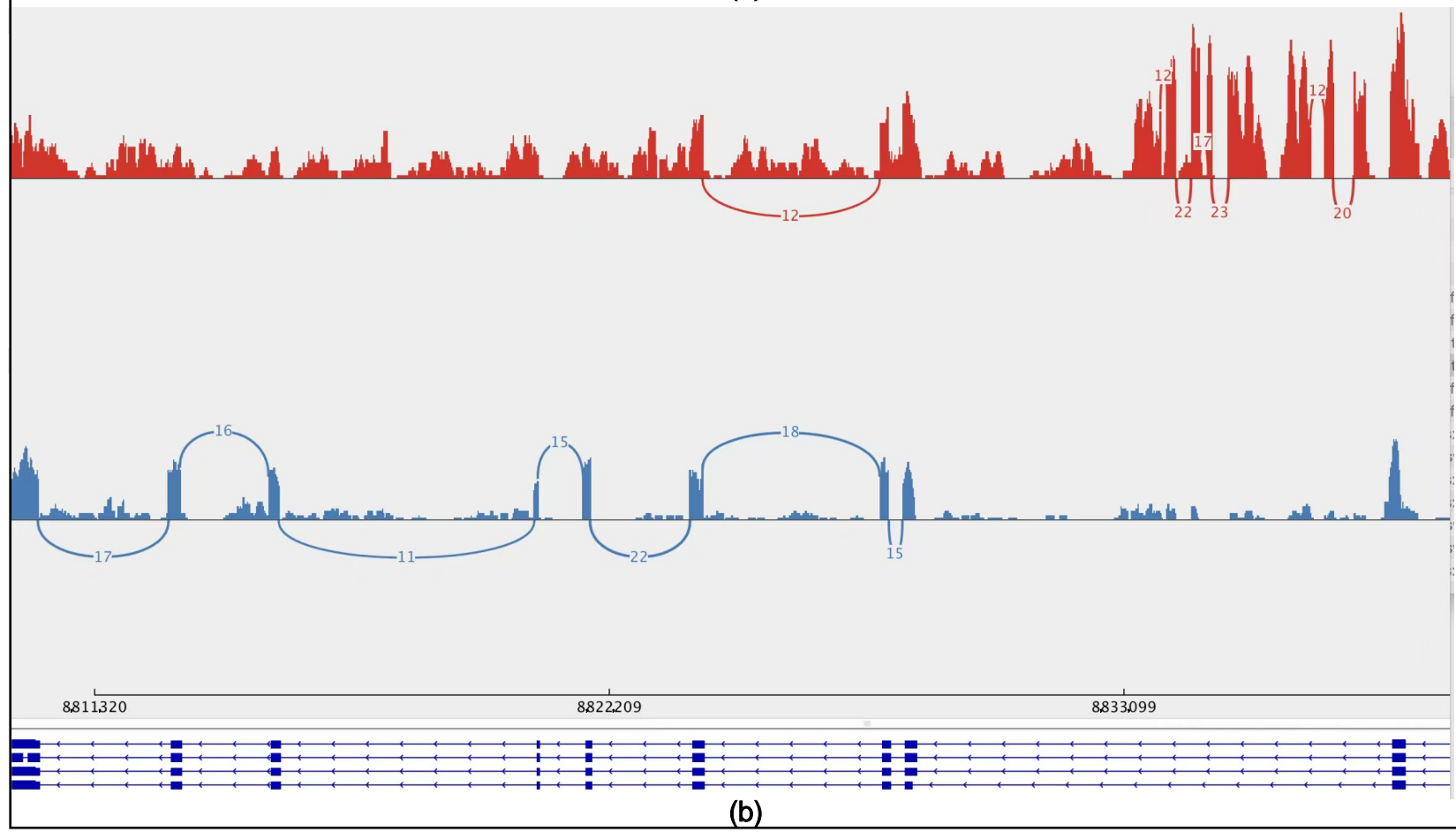

Supplement: Supplementary file 1 [file genes-16-00187-s001.zip › Supplemental Figure S3.pdf]

# Worker and Drone Novel Gene Expression Levels

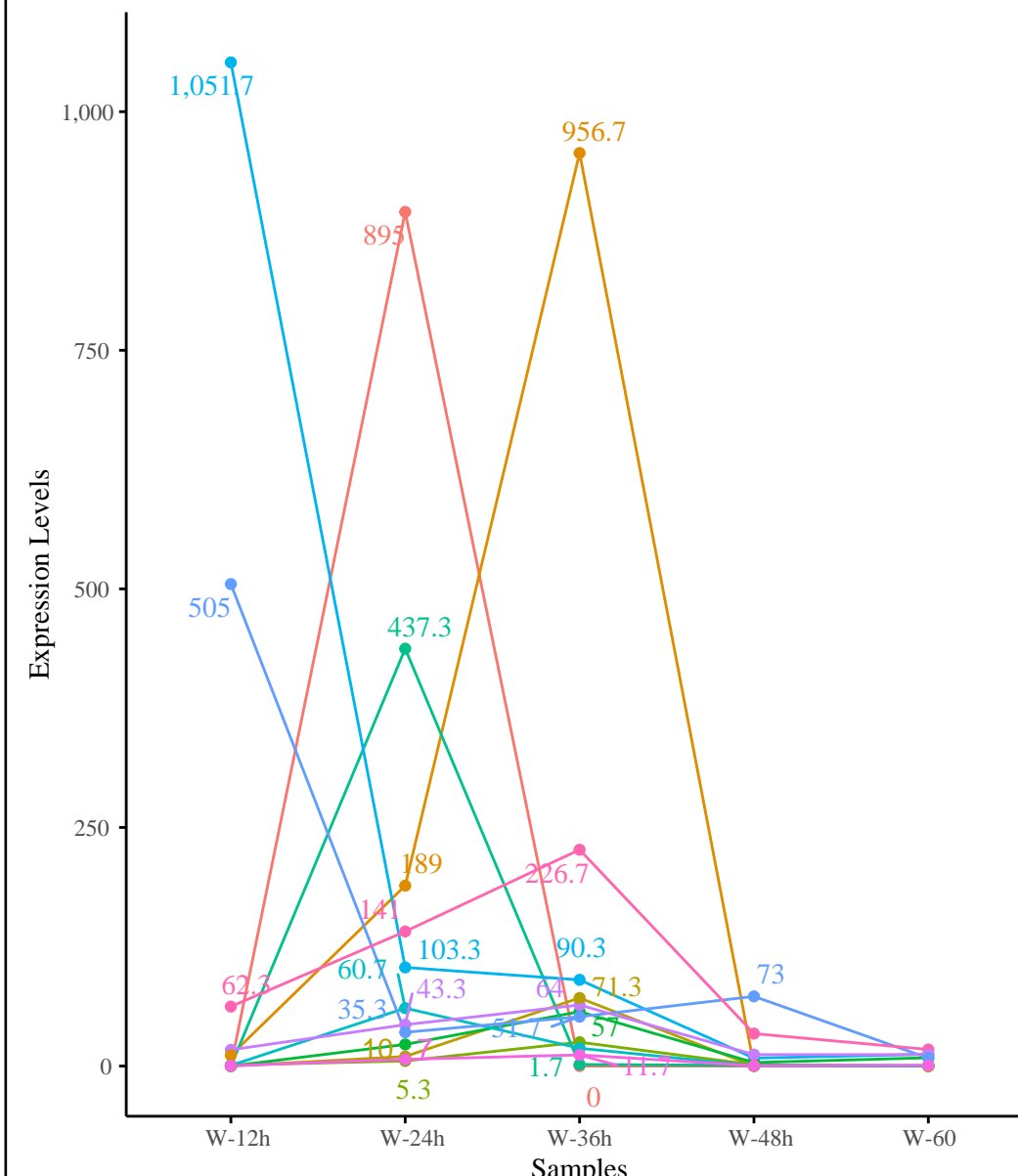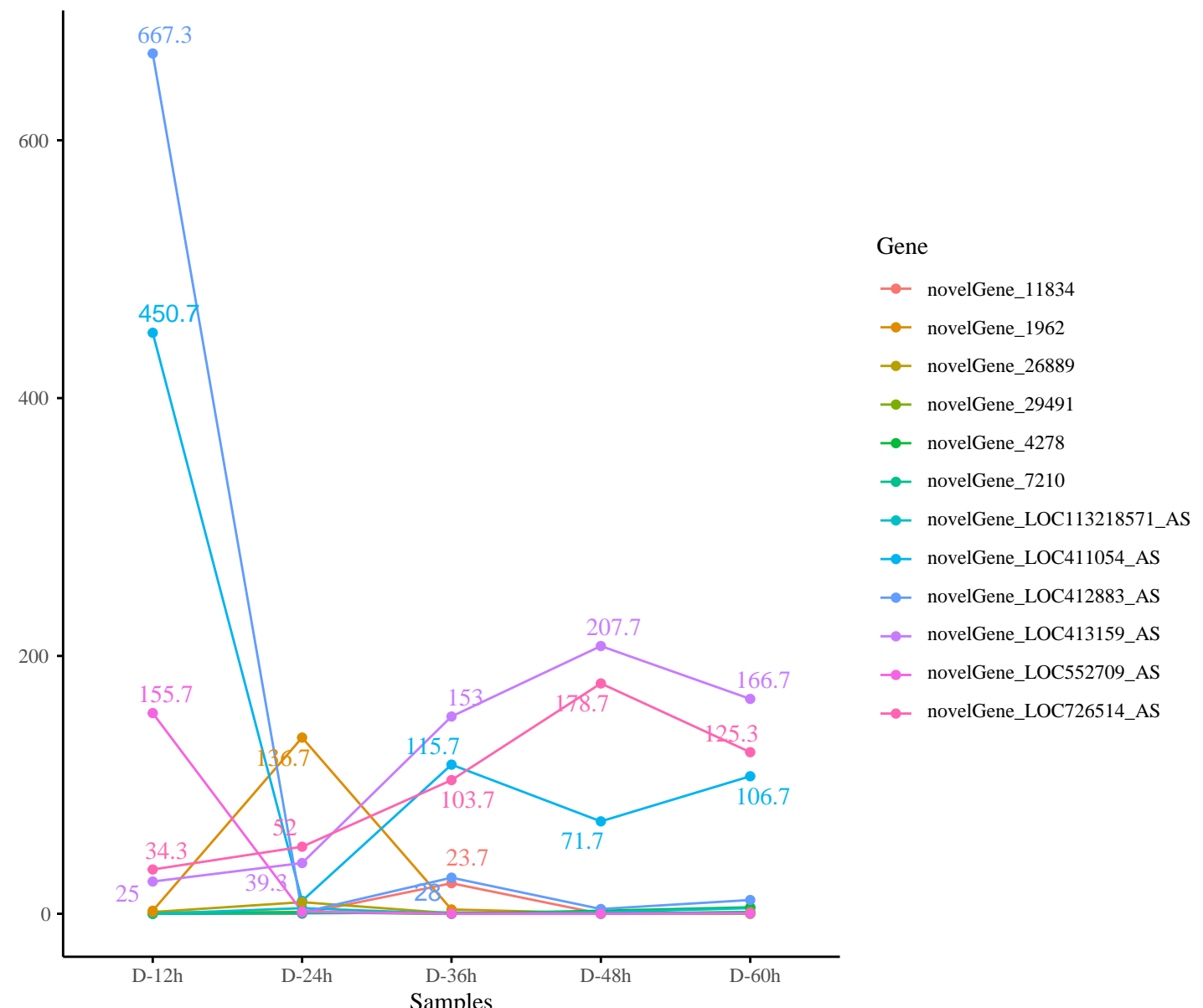

Supplement: Supplementary file 1 [file genes-16-00187-s001.zip › Supplemental Figure S4.pdf]

Line Plot of Group Means for Csd, Fem, and Dsx Genes in Workers and Drones

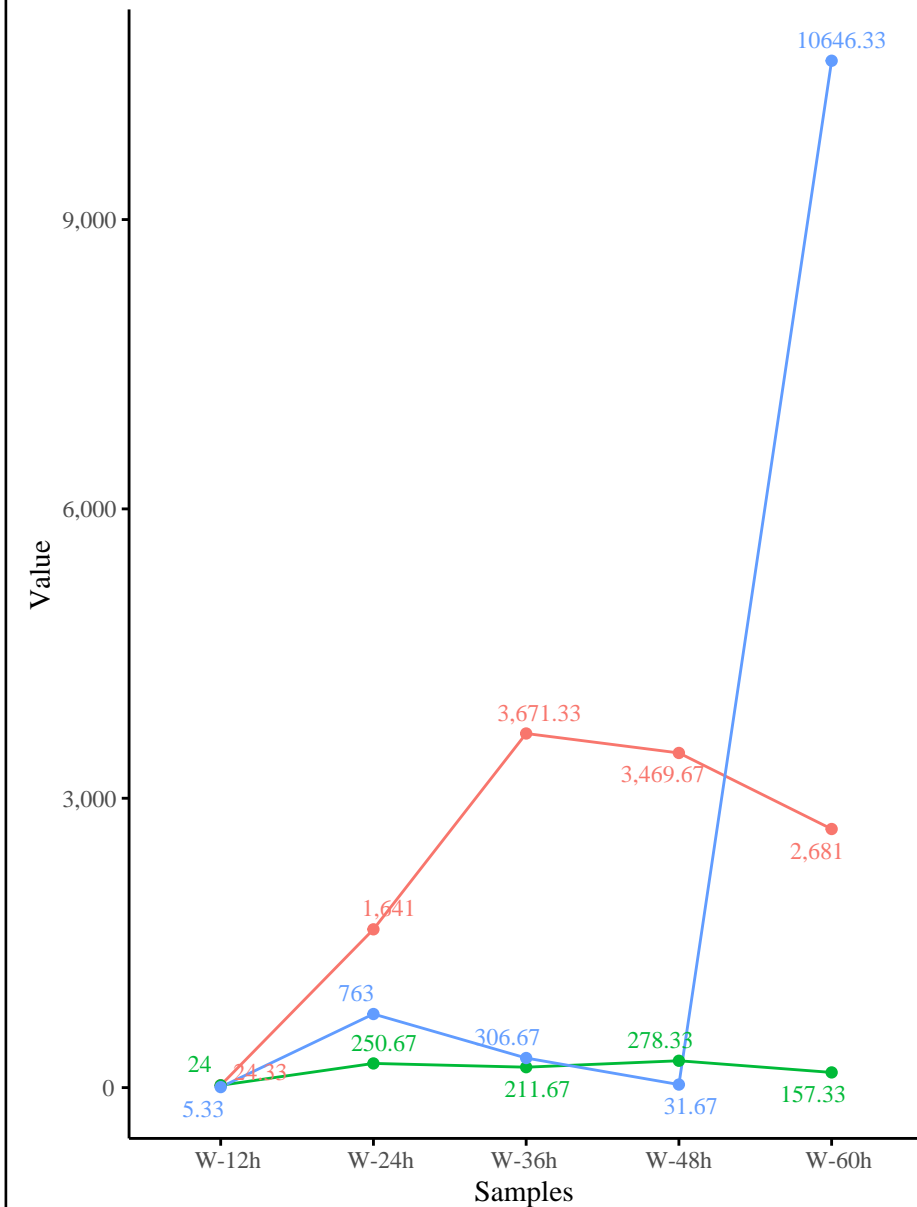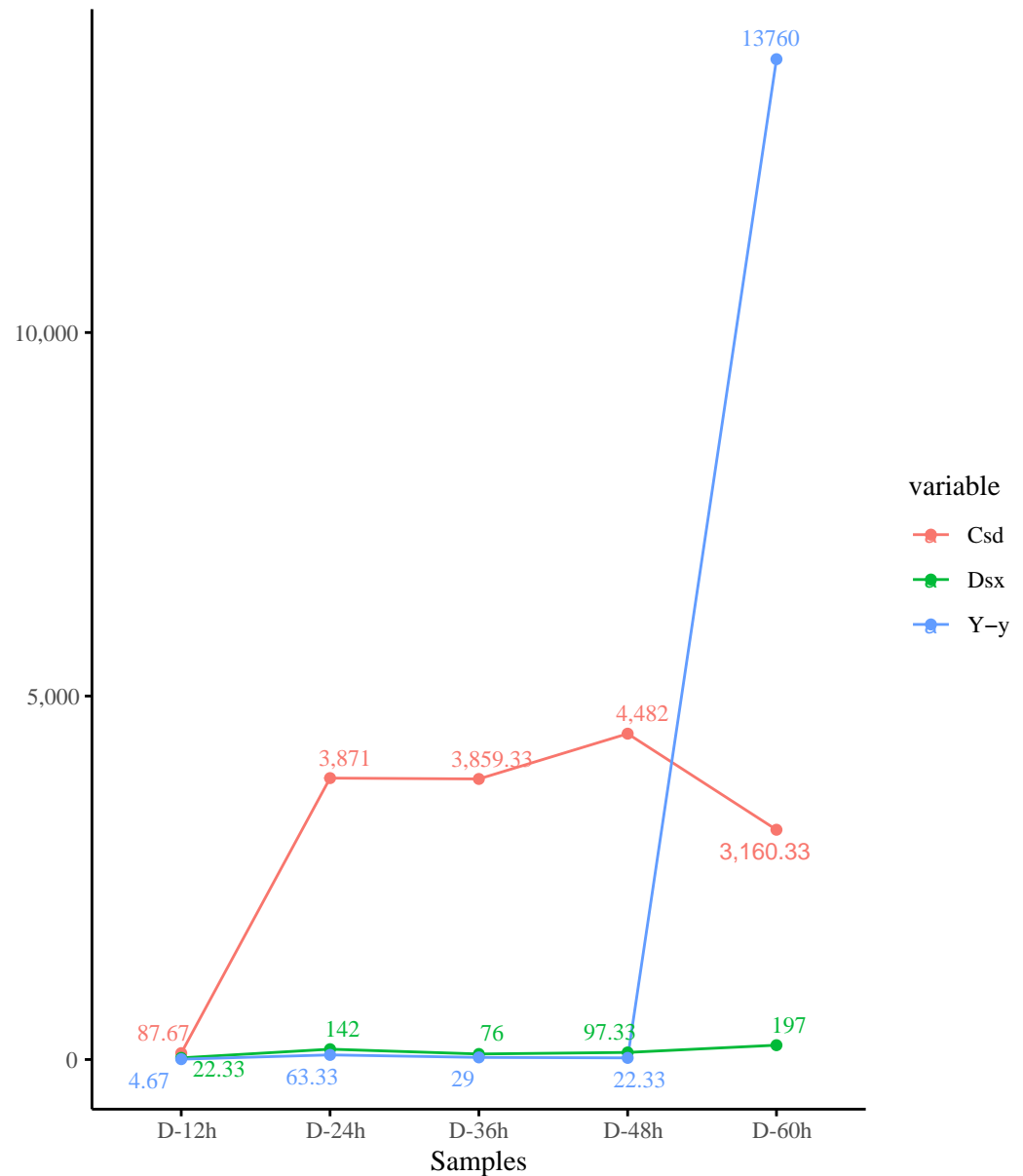

Supplement: Supplementary file 1 [file genes-16-00187-s001.zip › Supplemental Figure S5.pdf]
